# Light‐Regulation of Zeaxanthin Epoxidase in View of the Central Role of Zeaxanthin for Photoprotection

**DOI:** 10.1111/ppl.70617

**Published:** 2025-10-29

**Authors:** Peter Jahns, Stephanie Bethmann

**Affiliations:** ^1^ Photosynthesis and Stress Physiology of Plants Heinrich‐Heine‐University Düsseldorf Düsseldorf Germany

## Abstract

Plants are frequently exposed to fluctuating light conditions and, as a consequence, to variable photo‐oxidative stress. Efficient and flexible photoprotection is therefore essential for the fitness of plants in the field. The xanthophyll zeaxanthin, which is formed in high light from violaxanthin in the xanthophyll cycle, contributes to photoprotection in the thylakoid membrane at different levels, including the dissipation of excess light energy. Permanent high levels of zeaxanthin are known to compromise photosynthetic efficiency. It is thus of high importance to keep the amount of zeaxanthin at an optimal level in response to the growth light conditions. The zeaxanthin epoxidase, which reconverts zeaxanthin to violaxanthin, has been shown to be central to balancing the zeaxanthin amount in the thylakoid membrane. This review summarizes the recent advances in the understanding of the light regulation of zeaxanthin epoxidase in the context of the function of zeaxanthin in plant photoprotection.

## 1 Introduction

The conversion of light energy into chemical energy is the central process of the light reactions of photosynthesis (Renger 2010; Nelson and Junge 2015). This involves the absorption of light energy (by the antenna pigments of both photosystems) and its conversion into redox energy (through charge separation in both photosystems), which finally drives electron and proton transport and, by that, the formation of NADPH and ATP required for biomass production.

### 1.1 Efficient Light‐Harvesting Requires Efficient Photoprotection

Efficient utilization of light energy under limiting light availability is essential for the competitiveness and survival of plants in the field, particularly in dense stands. Hence, the two photosystems are equipped with light‐harvesting antenna complexes, which allow efficient photosynthesis under low light intensities. However, under natural fluctuating light intensities, this pivotal efficient light‐harvesting capacity frequently results in the absorption of excess light energy, which cannot be utilized in photosynthesis, bearing the risk of photo‐oxidative damage due to the formation of reactive oxygen species (ROS). To minimize the level of high‐light‐induced ROS formation, photosynthetic organisms have evolved different strategies, including (i) the reduction of light absorption, for example, by chloroplast movement (Wada et al. 2003) or the reduction of the photosystem II (PSII) antenna size (Walters 2005), (ii) the adjustment of photosynthetic electron transport (Kramer et al. 2004; Tikkanen et al. 2012; Schoettler and Toth 2014; Schumann et al. 2017), (iii) the dissipation of excitation energy as heat (= non‐photochemical quenching, NPQ) (Müller et al. 2001; Jahns and Holzwarth 2012; Ruban et al. 2012; Bassi and Dall'Osto 2021), and (iv) the detoxification of ROS by antioxidants and antioxidative enzymes (Moller et al. 2007; Foyer and Noctor 2011; Dumanovic et al. 2021). Together, these different strategies form a complex photoprotective network, which provides the basis for the proper short‐ and long‐term acclimation of plants to different light environments.

### 1.2 Zeaxanthin Contributes to Photoprotection at Different Levels

The xanthophyll zeaxanthin (Zx), which is formed in the so‐called xanthophyll cycle (Yamamoto et al. 1962; Jahns et al. 2009), serves central photoprotective functions in plants and algae (Demmig‐Adams and Adams 2006; Jahns and Holzwarth 2012). In the lipid phase of the thylakoid membrane, Zx acts as an antioxidant (Havaux and Niyogi 1999; Havaux et al. 2007) and modulates the stability and fluidity of the membrane (Havaux 1998). Moreover, Zx essentially contributes to different processes of non‐photochemical quenching (NPQ) of excess excitation energy in PSII, such as the energy‐dependent quenching qE (Horton et al. 2005), the Zx‐dependent quenching qZ (Nilkens et al. 2010), or the photoinhibitory quenching qI (Aro et al. 1993; Bethmann et al. 2019). Based on these different functions, Zx is involved in photoprotection at different time scales and under different light stress conditions. Proper short‐ and long‐term acclimation to different, often fluctuating light intensities is vital for plants to ensure maximum photoprotection under high light and to avoid unfavorable dissipation of excitation energy under low light conditions. The latter is particularly important because full activation of NPQ processes and accumulation of high levels of Zx in the thylakoid membrane under light‐limiting conditions have a negative impact on photosynthetic efficiency (Kromdijk et al. 2016; Garcia‐Molina and Leister 2020). Consequently, the amount of Zx in the thylakoid membrane and the dynamics of Zx synthesis and reconversion are critical determinants of the efficiency of photoprotection. Therefore, proper regulation of the Zx amount in the thylakoid membrane is required in the whole range of light intensities to optimize photosynthetic efficiency (under low light) and photoprotection (under high light).

### 1.3 Basic Features of the Xanthophyll Cycle Reactions

**FIGURE 1:**
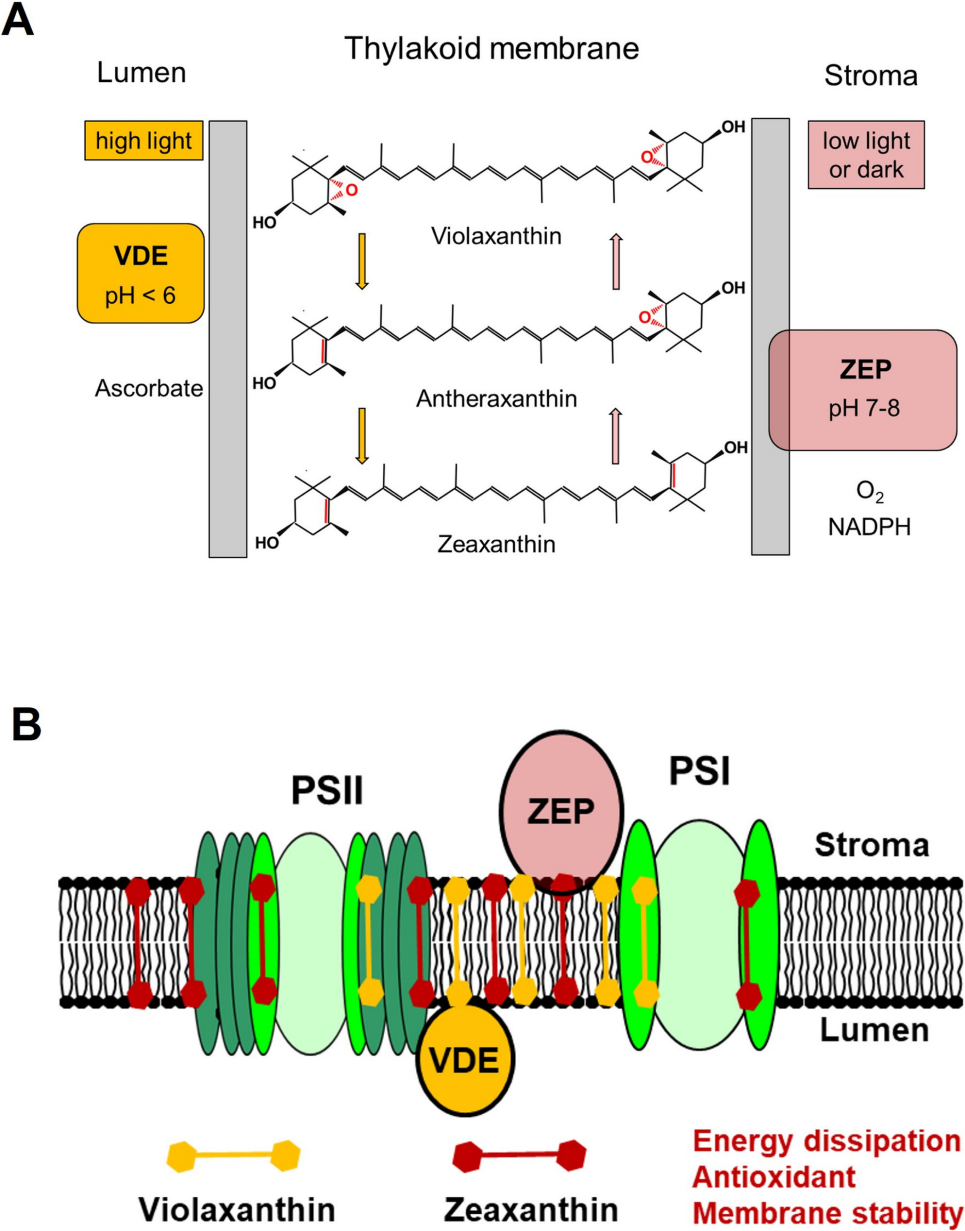
The xanthophyll cycle reactions. (A) Basic reactions and the required cofactors. (B) Localization of the substrates and the xanthophyll cycle enzymes in the thylakoid membrane. PSII, PSI = photosystem II and I; VDE = violaxanthin de‐epoxidase; ZEP = zeaxanthin epoxidase.

Zx is formed from violaxanthin (Vx) via the intermediate antheraxanthin (Ax) in the de‐epoxidation reactions of the xanthophyll cycle (Yamamoto et al. 1962; Jahns et al. 2009) (Figure 1). This reaction is catalyzed by the thylakoid lumen‐localized enzyme Vx de‐epoxidase (VDE), whose activity is regulated by the thylakoid lumen pH (Hager and Holocher 1994). VDE becomes activated at pH values below about 6.2–6.5 (Hager 1969; Pfündel and Dilley 1993), so that Zx is only formed under conditions when photosynthetic electron transport becomes light saturated. The reconversion of Zx to Vx is catalyzed by the Zx epoxidase (ZEP), which is localized in the chloroplast stroma (Bouvier et al. 1996; Schwarz et al. 2015). ZEP activity shows a less pronounced pH dependence with a maximum at about pH 7.5 (Siefermann and Yamamoto 1975), in accordance with the physiological pH range of the chloroplast stroma. Maximum ZEP activity has been determined under low light or in darkness under both in vivo (Reinhold et al. 2008) and in vitro (Siefermann and Yamamoto 1975; Holzmann et al. 2022) conditions.

The impact of the Zx amount in the thylakoid membrane on photoprotection and photosynthetic efficiency is supported by the phenotypes of the two xanthophyll cycle mutants, the Zx‐deficient *npq1* mutant (no VDE activity) and the Zx‐accumulating *npq2* mutant (no ZEP activity) (Niyogi et al. 1998). The absence of Zx leads to a strongly increased high light sensitivity, which is predominantly related to qE‐independent functions of Zx, as has been derived from the comparison of the high light responses of the qE‐deficient *npq4* mutant and the qE‐ and Zx‐deficient *npq4npq1* double mutant (Havaux and Niyogi 1999). In contrast, the permanent presence of high Zx amounts results in lower PSII efficiency and reduced growth under non‐saturating light conditions (Kalituho et al. 2007).

### 1.4 Zeaxanthin Amount and Photosynthetic Efficiency

Photosynthetic efficiency has further been supposed to be limited by the kinetics of NPQ induction and relaxation (in particular of qE and qZ), especially under fluctuating light conditions, which frequently occur under natural conditions on partly cloudy days (Zhu et al. 2004; Murchie and Niyogi 2011). Since high amounts of Zx accelerate the induction of NPQ, but decelerate the relaxation of NPQ (Niyogi et al. 1998), acceleration of Zx synthesis and reconversion has been proposed as a promising target to improve photosynthetic efficiency. Consequently, bioengineering of the dynamics of NPQ induction and relaxation has been applied to improve photosynthesis under fluctuating light conditions (Kromdijk et al. 2016; Garcia‐Molina and Leister 2020; Lehretz et al. 2022). In fact, over‐expression of the Arabidopsis ( *Arabidopsis thaliana* ) genes coding for VDE, PsbS and ZEP (VPZ) in tobacco ( *Nicotiana tabacum* ; Kromdijk et al. 2016), Arabidopsis (Garcia‐Molina and Leister 2020), potato ( *Solanum tuberosum* ; Lehretz et al. 2022) and soybean ( *Glycine max* ; De Souza et al. 2022) induced faster NPQ induction and/or faster NPQ relaxation. However, a positive impact on biomass production in these VPZ lines was only determined for tobacco and soybean plants, but not for Arabidopsis and potato plants.

## 2 Determinants of the Amount of Zeaxanthin in the Thylakoid Membrane

The amount of Zx that accumulates in the thylakoid membrane is determined by two factors: (i) the total amount of xanthophyll cycle pigments (= VAZ pool size) and (ii) the steady‐state of the de‐epoxidation state (DEPS = [Zx + 0.5Ax]/[Vx + Ax+Zx]) of the xanthophyll cycle pigments.

### 2.1 The VAZ Pool Size

**FIGURE 2:**
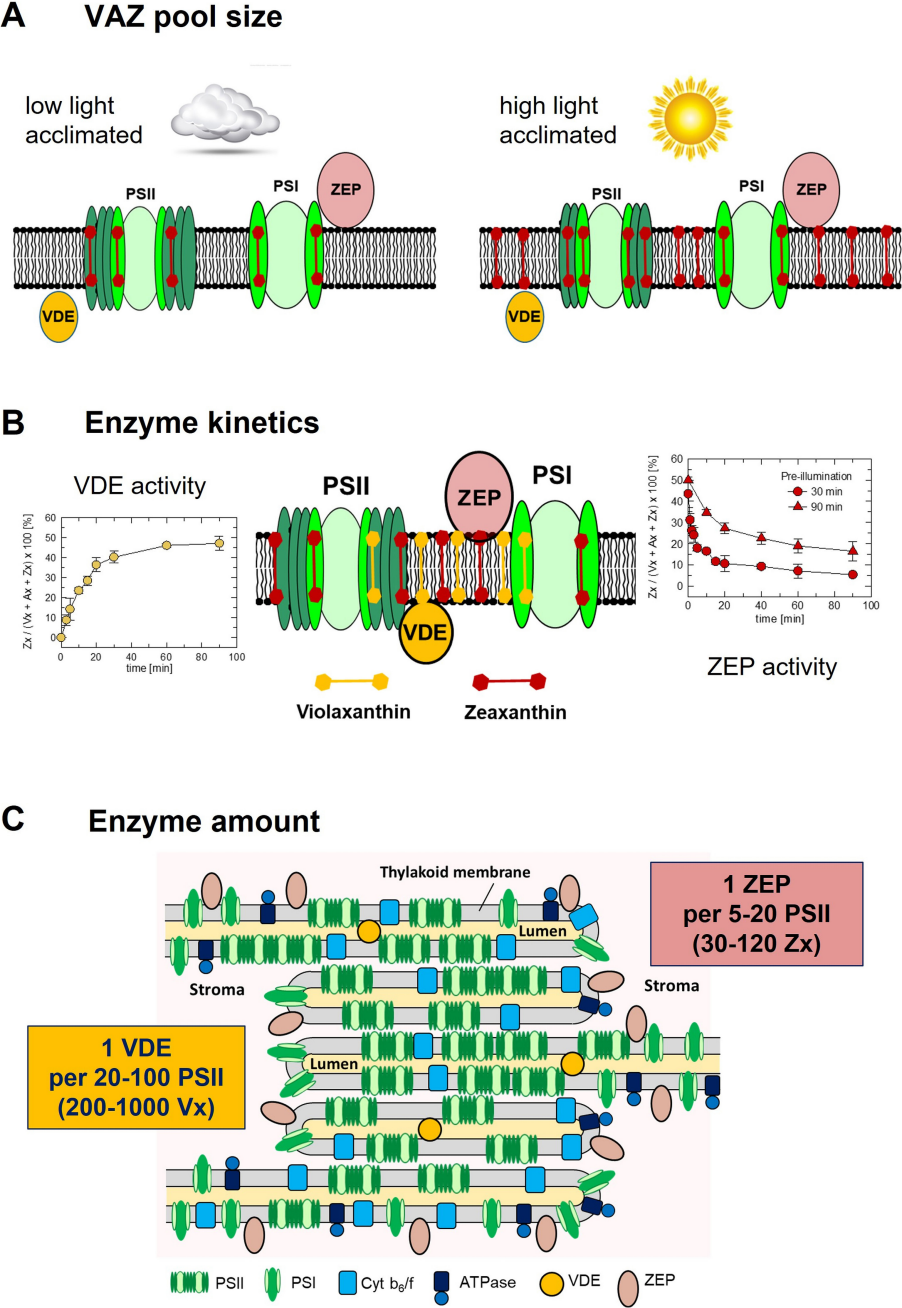
Determinants of the amount of zeaxanthin in the thylakoid membrane. (A) The impact of growth light conditions on the amount of xanthophyll cycle pigments in the thylakoid membrane (= VAZ pool size). (B) Kinetics of xanthophyll conversion. For VDE activity, the graph illustrates the formation of zeaxanthin in Arabidopsis leaves in response to a dark–light transition (900 μmol photons m^−2^ s^−1^). For ZEP activity, the decrease of the zeaxanthin content in response to a light–dark transition [pre‐illumination at 900 μmol photons m^−2^ s^−1^ for either 30 min (circles) or 90 min (triangles)] is shown. (**C**) Localization and amount of the xanthophyll cycle enzymes. The shown amounts of PSII (50), VDE (3) and ZEP (12) roughly approximate the ratios that have been estimated from proteomics data. ATPase = ATP synthase; Cyt b_6_/f = Cytochrome b_6_/f complex; PSII, PSI = photosystem II and I; VDE = violaxanthin de‐epoxidase; ZEP = zeaxanthin epoxidase.

The VAZ pool size varies among different species, typically in the range from about 30 to 100 VAZ per 1000 Chl (Demmig‐Adams et al. 2012; Bethmann et al. 2023) and is particularly adjusted to the environmental growth conditions (Figure 2A). A larger VAZ pool size is typically found in evergreen species compared to seasonal species (Demmig‐Adams et al. 2012), in sun plants compared to shade plants (Thayer and Björkman 1990; Demmig‐Adams 1998; Matsubara et al. 2009), and in plants acclimated to high light compared to those acclimated to low light (Bailey et al. 2004; Schumann et al. 2017). Comparison of species with different VAZ pool sizes that were grown under similar light conditions (Bethmann et al. 2019) or acclimated to different growth light intensities (Bethmann et al. 2023) showed that a larger VAZ pool is accompanied by an increased resistance against photoinhibition. This might possibly be related to the presence of a substantial amount of non‐protein‐bound pool of VAZ pigments in plants with a larger VAZ pool size (Bethmann et al. 2019; Bethmann et al. 2023) (Figure 2A). The VAZ pool size can thus be considered a critical parameter for protection against long‐lasting high light stress. The amount of VAZ pigments in the thylakoid membrane is likely determined by the activity of the β‐carotene hydroxylase (named CHYB, BCH or CRTZ), which converts β‐carotene to Zx (Sun et al. 1996), as can be derived from the increased VAZ pool size in *CHYB* overexpressing plants (Davison et al. 2002; Wu et al. 2012; Zhao et al. 2014). Strikingly, overexpressing Arabidopsis CHYB (AtCHYB) in Arabidopsis (Davison et al. 2002) and *Eustoma grandiflorum* Shinn (Wu et al. 2012) was found to increase the resistance towards high light stress and its overexpression in tobacco increased drought tolerance (Zhao et al. 2014). Hence, genetical engineering of the VAZ pool might be promising to improve the high light and drought resistance of crop plants. However, the increase of the VAZ pool size in *CHYB*‐overexpressing Arabidopsis plants (Davison et al. 2002) was accompanied by retarded NPQ dynamics, affecting both NPQ induction and NPQ relaxation (Johnson et al. 2008). Although the maximum NPQ capacity was not altered in such plants, the slower NPQ dynamics can be expected to compromise their photoprotective capacity and/or photosynthetic efficiency under natural fluctuating light or low light conditions (Zhu et al. 2004; Johnson et al. 2008). A large VAZ pool size might thus be unfavorable under non‐saturating light conditions. However, comparison of the photosynthetic performance of species with different VAZ pool sizes under varying growth light conditions did not identify a general disadvantage of an increased VAZ pool size under low light conditions (Bethmann et al. 2023), indicating that species‐specific properties might compensate for possible drawbacks of a larger VAZ pool size with respect to photosynthetic efficiency.

### 2.2 The De‐Epoxidation State (DEPS) of the VAZ Pigments

In addition to the VAZ pool size, the DEPS of the VAZ pool determines the amount of Zx in the thylakoid membrane. Independent of the VAZ pool size, the DEPS at a given light intensity is determined by the equilibrium of the rates of xanthophyll conversion catalyzed by the two xanthophyll cycle enzymes, VDE and ZEP. The xanthophyll conversion rates depend on three factors: (i) the specific enzyme activities of VDE and ZEP, (ii) the amount of the two proteins, and (iii) the availability of the substrate, Vx and Zx, respectively.

All three factors are influenced by the localization of VDE and ZEP. While VDE localizes to the thylakoid lumen, ZEP is localized in the chloroplast stroma. Both enzymes are soluble proteins binding to the membrane surface. VDE binds reversibly to the thylakoid membrane in dependence on the pH of the thylakoid lumen. VDE binding is induced at pH values below about 6.5 (Hager and Holocher 1994). Binding of VDE to the membrane is likely accompanied by the formation of dimers (Arnoux et al. 2009). In contrast to that, ZEP is constitutively associated with the thylakoid membrane through hydrophobic interactions (Schwarz et al. 2015). In some species, such as Arabidopsis or tobacco, a fraction of ZEP protein may be present in the chloroplast stroma (Schwarz et al. 2015; Bethmann et al. 2019). With respect to the xanthophyll conversion rates, one important difference between VDE and ZEP is that VDE has access to all regions of the thylakoid membrane—due to its mobility in the thylakoid lumen—whereas ZEP is restricted to the stroma‐exposed regions of the membrane, without access to the inner part of the grana stacks, similar to PSI (Figure 2C). However, comparison of xanthophyll conversion in separated stroma and grana fractions of thylakoid membranes isolated from pea ( *Pisum sativum* ) showed similar activities in both compartments for both VDE and ZEP (Färber and Jahns 1998). Thus, ZEP protein seems to be evenly distributed among stroma membranes and stroma‐exposed regions of grana membranes, whereas VDE can be expected to be randomly distributed in the lumen among stroma and grana membranes.

#### 2.2.1 Specific Enzyme Activities of VDE and ZEP

As judged from the net changes of the Zx content in leaves (in Figure 2B shown for Arabidopsis), the rates of the conversion of Vx to Zx in high light and of the reconversion of Zx to Vx (in low light or darkness) occur at a rather similar time scale, at least when both enzymes work at maximum rates. In general, Zx synthesis by VDE at saturating light intensities has been estimated to be about 5‐ to 10‐fold faster than Zx reconversion to Vx by ZEP (Härtel et al. 1996), ensuring that high DEPS values and thus maximum photoprotection are reached under saturating light conditions. It should be noted, however, that these estimates were based on the assumption that Zx to Vx reconversion—and hence ZEP activity—is negligible during the initial phase after a dark to light transition (Härtel et al. 1996). This assumption, however, may not be justified, since recent work showed that ZEP is fully active in the light‐ and dark‐acclimated state under in vitro conditions (Holzmann et al. 2022), and Zx to Vx reconversion was shown to occur at high rates even after only 5 min of pre‐illumination in intact leaves as well (Nilkens et al. 2010; Kress and Jahns 2017). Moreover, Zx formation in intact leaves was found to be clearly accelerated at saturating and non‐saturating light intensities when ZEP activity was inhibited by salicylaldoxime (Hoang et al. 2020). It can thus be assumed that the maximum VDE activity—as given at a lumen pH < 5.8, and thus under saturating light conditions—might be even more than 5–10‐fold faster than the maximum ZEP activity. At non‐saturating light intensities, however, when the lumen pH does not drop to values below pH 6.0 and thus does not lead to full activation of VDE, ZEP activity may overcome VDE activity, so that the DEPS under steady state conditions is rather low (Adams and Demmig‐Adams 1992; Jahns 1995) and photosynthetic efficiency is thus not limited by Zx‐dependent dissipation of excitation energy. Light‐dependent adjustment of the steady‐state DEPS is therefore essential to balance photoprotection under high light and photosynthetic efficiency under low light. This adjustment is provided by the light regulation of the activity of both enzymes, VDE and ZEP. Whereas VDE is strictly regulated by the lumen pH, which is a direct measure of the saturation of photosynthetic electron transport, ZEP activity becomes stepwise downregulated in response to prolonged high light stress (Reinhold et al. 2008) (see also Figure 2B). The light regulation of ZEP activity will be addressed in more detail below.

#### 2.2.2 The Amount of VDE and ZEP Protein

In addition to the specific activities, the amount of VDE and ZEP can be expected to impact the DEPS and thus the Zx amount in the thylakoid membrane. Early studies on mutants overexpressing either VDE or ZEP protein provided only limited data on the influence of the enzyme amount on the kinetics of xanthophyll conversion and Zx accumulation. Overexpression of VDE in tobacco (Gao et al. 2010) and tomato (Han et al. 2010) induced slightly increased Zx accumulation, which led to only slightly increased NPQ and reduced photoinhibition of PSII. In contrast, overexpression of ZEP in tomato resulted in decreased Zx accumulation and NPQ, but in increased photoinhibition of PSII (Wang et al. 2008). Aiming at the improvement of photosynthetic efficiency, more recent work applied over‐expression of both VDE and ZEP (in addition to PsbS) to speed up the NPQ dynamics (Kromdijk et al. 2016). Indeed, these VPZ plants showed a reduced DEPS under both constant light and fluctuating light (Kromdijk et al. 2016), supporting the view that the amounts of VDE and ZEP are important for the control of the DEPS and thus the Zx amount in the thylakoid membrane.

Using the VPZ lines from tobacco (Kromdijk et al. 2016) and Arabidopsis (Garcia‐Molina and Leister 2020), the impact of increased amounts of VDE and ZEP on xanthophyll conversion has been studied in more detail in response to moderate high light treatment, that is, 30 min illumination at light intensities ranging from 100 to 2000 μmol photons m^−2^ s^−1^ (Küster et al. 2023). Although both enzymes were enriched to a similar extent by at least a factor of 5 compared to WT plants, an increase in the VDE activity in VPZ lines was not obvious from the kinetics of Vx to Zx conversion in either species, while a clearly higher ZEP activity was observable for VPZ lines of both species. This suggests that the amount of ZEP but not the amount of VDE is a critical determinant of the equilibrium of the DEPS of the xanthophyll cycle pigments under moderate high light conditions, at least in vascular plants. Obviously, the amount of ZEP protein is adjusted to a level that prevents rapid reconversion of Zx to Vx at saturating light conditions (Küster et al. 2023). This adjustment of the ZEP level strongly supports the physiological necessity to keep Zx reconversion to Vx and hence ZEP activity rather low even under non‐stressful light conditions. However, the missing impact of an increased VDE content on Zx formation found in vascular plants does not apply to the microalga *Nannochloropsis oceanica*, where an increase in the amount of both, VDE and ZEP, was recently shown to result in accelerated conversion of Vx to Zx and Zx to Vx, respectively (Michelberger et al. 2025). Obviously, both reactions of the xanthophyll cycle run in this microalga at submaximal rates. This is likely related to the very large VAZ pool size in *N. oceanica* (500 VAZ per 1000 Chl; Michelberger et al. 2025), which is about 5–10‐fold larger than that of vascular plants (30–100 VAZ per 1000 Chl).

#### 2.2.3 The Substrate Availability

Plants with large VAZ pool sizes typically show higher maximum DEPS than plants with smaller VAZ pool sizes (Thayer and Björkman 1990; Bethmann et al. 2019), which further increases the Zx amount that can accumulate in the thylakoid membrane in response to high light in plants with a large VAZ pool. This higher convertibility of Vx to Zx is likely related to an increase in non‐protein‐bound or loosely bound Vx. It is known from earlier in vitro studies (Jahns et al. 2001; Wehner et al. 2004; Wehner et al. 2006) that the non‐convertible fraction of the VAZ pool depends on the xanthophyll binding site in the different antenna proteins. In general, Vx bound to the L2 site of LHC proteins is supposed to be less easily and less rapidly available for conversion by VDE, while Vx bound to the V1 site—as in trimeric LHCII—and non‐protein Vx are fully and rapidly convertible to Zx (Jahns et al. 2001). Both enzymes of the xanthophyll cycle, VDE and ZEP, are supposed to convert non‐protein‐bound substrates that are available in the lipid phase of the thylakoid membrane. Since both enzymes bind to the thylakoid membrane and are rather immobile, the release and/or the diffusion of the substrate is rate‐limiting for xanthophyll conversion (Macko et al. 2002; Küster et al. 2023). Hence, loosely bound or non‐protein‐bound xanthophylls are most suitable for rapid and efficient conversion and will thus lead to the accumulation of loosely bound or non‐protein‐bound Zx under high light stress conditions. Plants with a large VAZ pool size will thus contain a larger fraction of Zx, which may function as an antioxidant or membrane stabilizer in the lipid phase of the membrane. Such functions of Zx in the lipid phase of the thylakoid membrane might be crucial determinants of the increased high‐light resistance of plants with a large VAZ pool size.

## 3 Down‐Regulation of ZEP Activity in Response to High Light Stress

Whereas the amount of ZEP protein limits Zx reconversion to Vx under non‐stressful light conditions, the down‐regulation of ZEP activity is required in response to high light stress to retain high amounts of Zx under such unfavorable conditions. Three principal ways of ZEP inactivation have been proposed in the literature: (i) phosphorylation (Xu et al. 1999; Kim et al. 2017; Hoang et al. 2020), (ii) redox regulation (Naranjo et al. 2016; Da et al. 2017) and (iii) ROS modification (Reinhold et al. 2008; Bethmann et al. 2019). While phosphorylation and TRX‐mediated redox regulation can be expected to be reversible processes, ROS‐induced inactivation likely represents an irreversible inactivation mechanism.

### 3.1 Phosphorylation‐Mediated Regulation of ZEP Activity

**FIGURE 3:**
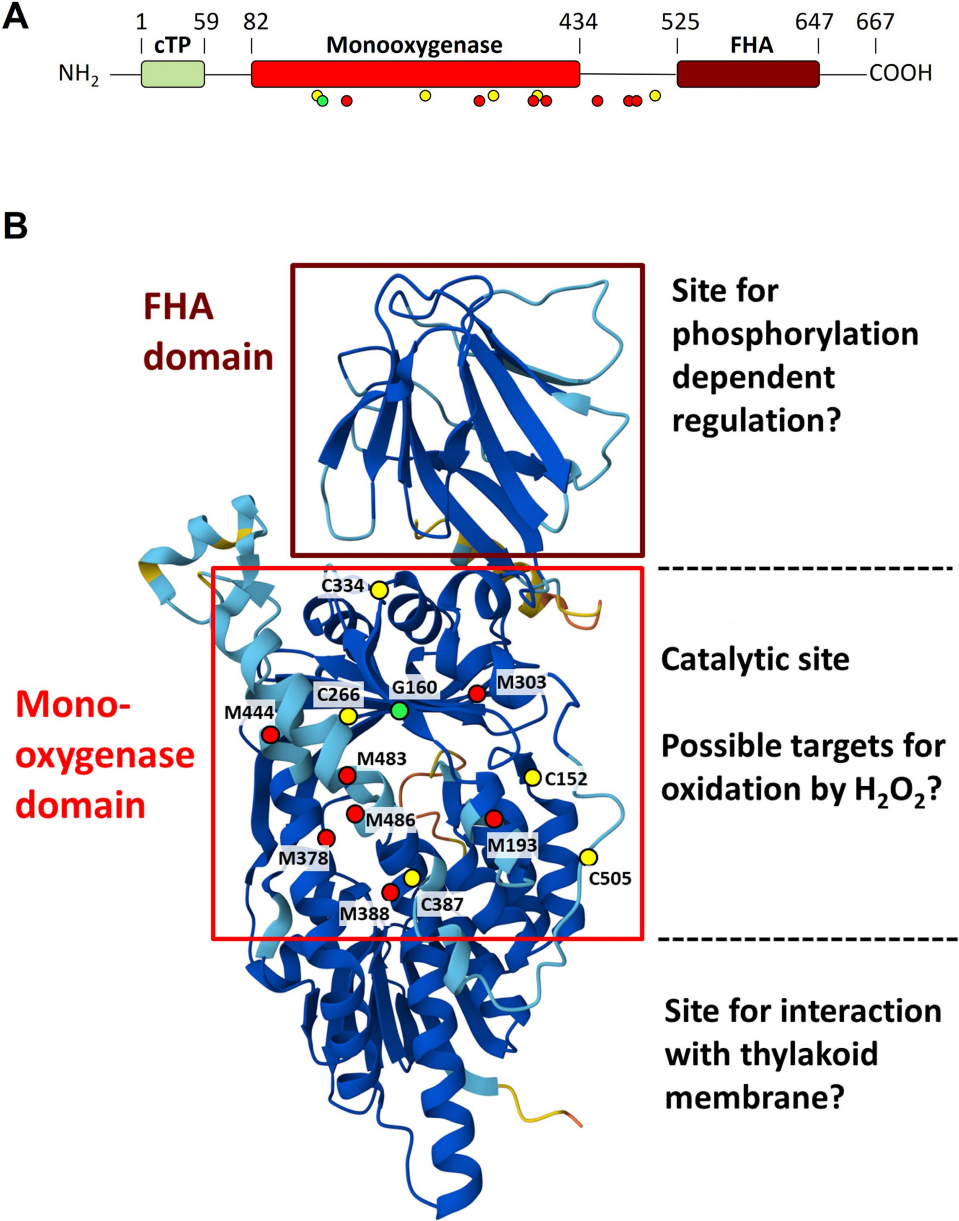
Structure of ZEP protein from Arabidopsis. (A) Schematic alignment of the ZEP protein. (B) Predicted structure of the ZEP protein. The enzyme is supposed to be composed of a chloroplast transit peptide (cTP), a central monooxygenase domain and a C‐terminal fork head associated (FHA) domain. The colored dots in (A) and (B) highlight seven conserved Met residues (M, red), five conserved Cys residues (C, yellow) and one residue (glycine G160, green), whose mutation led to inactivation of ZEP (Niyogi et al. 1998; Barrero et al. 2005). The central monooxygenase domain (red box) contains an ATP and FAD binding motif (Marin et al. 1996) and three lipocalin motifs (Bugos et al. 1998) and likely represents the active site. The structural model was taken from the AlphaFold Protein Structure Database (https://alphafold.ebi.ac.uk). For clarity, the first 70 amino acids of the N‐terminus containing the chloroplastic transit peptide were removed.

A possible role of phosphorylation in the regulation of ZEP activity has been derived from the finding that Zx epoxidation is retarded in leaves after infiltration with the phosphatase inhibitors NaF or Na_2_MoO_4_ (Xu et al. 1999). Phosphorylation of ZEP itself has not been proven so far but cannot be ruled out. However, the earlier observation that phosphorylation of LHCII and PSII core proteins correlates with down‐regulation of ZEP activity (Ebbert et al. 2001; Ebbert et al. 2005) makes it more likely that the phosphorylation of other proteins might trigger the down‐regulation of ZEP activity. It is well known that photoinhibition and repair of PSII involve the phosphorylation of PSII core proteins (Aro et al. 1993; Tikkanen and Aro 2012). The reported gradual down‐regulation of ZEP activity upon increasing photoinhibition of PSII (Reinhold et al. 2008) might thus indeed be linked to the phosphorylation of PSII core proteins along with photoinhibition of PSII. Related to this, the C‐terminal fork head associated (FHA) domain of ZEP (Figure 3) might play a critical role in the phosphorylation‐mediated regulation of ZEP activity, since FHA domains are known to recognize phosphoproteins (Chevalier et al. 2009). However, the importance of the FHA domain for the activity of ZEP remains to be clarified. The possible involvement of the high‐light‐induced phosphorylation of PSII core proteins has been challenged by the similar high‐light‐induced inactivation of ZEP in Arabidopsis wild‐type plants and the *stn7stn8* mutant (Reinhold et al. 2008), which is defective in the thylakoid protein kinases STN7 and STN8, which phosphorylate LHCII and PSII core proteins, respectively (Bonardi et al. 2005). However, this finding does not necessarily exclude the regulation of ZEP by high‐light‐induced protein phosphorylation. Alternatively, other kinases might be responsible for the phosphorylation of a protein acting as a regulator of ZEP activity, or the high‐light stress conditions applied in the work by Reinhold et al. (2008) rather induced ROS‐induced irreversible inactivation of ZEP instead of phosphorylation‐mediated reversible down‐regulation.

### 3.2 Redox Regulation of ZEP Activity

The idea of the redox regulation of ZEP activity was derived from two studies on mutants defective in either NADPH thioredoxin reductase C (NTRC) (Naranjo et al. 2016) or thioredoxin (TRX)‐m (Da et al. 2017), which both exhibited increased Zx levels in dark‐acclimated plants, indicating reduced ZEP activity. Both studies described the redox‐dependent formation of ZEP multimers/oligomers, suggesting that the reduction of ZEP through NTRC or TRX‐m may be required for full activation of ZEP. Hence, ZEP might be a classical redox‐modulated enzyme, requiring the reduction of a disulfide bridge for full activation, suggesting that specific cysteine (Cys) residues are essential for light‐dependent ZEP regulation. This would be in line with the hypothesis of earlier work, suggesting that the inactivation of ZEP by cadmium is related to the oxidation of a conserved Cys residue (Latowski et al. 2005), likely corresponding to C266 in Arabidopsis ZEP (Figure 3B). However, in the study with Arabidopsis *ntrc* mutants, formation of ZEP multimers in the oxidized state was only found for recombinant ZEP protein, while no changes in the redox state and no multimer formation of the native protein were detectable under in vivo conditions, in either the dark‐acclimated or light‐acclimated state (Naranjo et al. 2016). In agreement with this finding, a study with isolated spinach thylakoids showed that the ZEP is fully active in both the dark‐ and light‐acclimated state and thus not responsive to thiol modulation (Holzmann et al. 2022). This would exclude a classical light/dark regulation of ZEP activity by TRX. Nevertheless, the shown interaction of ZEP and TRX‐m (Da et al. 2017) suggests an impact of the TRX‐m redox state on ZEP function, possibly independent of the classical thiol modulation. It can be speculated that TRX‐m may be involved in modifying the interaction of ZEP with the thylakoid membrane or with other proteins in the chloroplast stroma.

### 3.3 ROS‐Induced Inactivation of ZEP

A very peculiar characteristic of ZEP regulation is the gradual down‐regulation of ZEP activity in response to increasing high light stress (Reinhold et al. 2008; Kress and Jahns 2017; Bethmann et al. 2019). In extreme cases, high light may lead to complete inactivation of ZEP, not only under controlled lab conditions (Bethmann et al. 2019; Bethmann et al. 2023) but also in the field, such as in over‐wintering species (Verhoeven et al. 1996; Demmig‐Adams et al. 2012). In both cases, inactivation of ZEP is correlated with the inactivation of PSII. Under severe conditions, ZEP protein is even degraded along with the D1 protein of PSII (Bethmann et al. 2019, Bethmann et al. 2023). It has been assumed that ROS have a central function in the inactivation of ZEP (Reinhold et al. 2008; Bethmann et al. 2019). The possible impact of different ROS on ZEP activity has been studied in isolated thylakoid membranes (Holzmann et al. 2022). ZEP activity was found to be sensitive to hydrogen peroxide (H_2_O_2_) but not to singlet oxygen. The study further supported the view that superoxide generated at the acceptor site of PSI does not accumulate to reasonable levels in presence of thylakoid‐associated superoxide dismutase. Hence, the high‐light‐induced inactivation of ZEP is likely based on the inactivation by hydrogen peroxide (Holzmann et al. 2022). The most likely targets for oxidation by hydrogen peroxide are the sulfur‐containing amino acids Cys and methionine (Met) (Moller et al. 2007). Related to the predicted structure of ZEP (Figure 3B), the majority of the conserved Cys and Met residues localize to the central mono‐oxygenase domain of ZEP. These residues represent the most likely targets for the proposed H_2_O_2_‐induced inactivation of ZEP.

The gradual down‐regulation of ZEP activity parallel to increasing photoinhibition of PSII ensures that Zx is retained after periods of extreme high light stress, suggesting that Zx has a pivotal photoprotective function during the repair cycle of damaged PSII or under unfavorable environmental conditions, such as during winter in evergreen plants. Interestingly, ZEP activity was shown to be inhibited by prolonged illumination also in the microalga *N. oceanica* (Michelberger et al. 2025). This suggests that the post‐translational down‐regulation of ZEP activity represents a common regulatory mechanism among plants and algae.

## 4 Conclusions

Zx serves central photoprotective functions in chloroplasts and the regulation of the amount of Zx in the thylakoid membrane is essential for the proper acclimation of plants to fluctuating light conditions. The limitation of ZEP activity in vascular plants is important at different time scales and different high‐light intensities. ZEP activity is adjusted in a way that efficient photoprotection by Zx is ensured at all light intensities, and that Zx is rapidly reconverted to Vx after moderate light stress, but retained after extreme high light stress. The retention of Zx is critical for photoprotection under a wide range of physiological conditions. Obviously, the amount of Zx in the thylakoid membrane is particularly controlled by the amount of ZEP protein under non‐stress conditions and the light‐dependent down‐regulation of ZEP activity under high light stress. In response to extreme high light conditions, ZEP might become completely inactivated or even degraded. The post‐translational inactivation of ZEP is likely mediated by phosphorylation and/or hydrogen peroxide. ROS‐induced down‐regulation and/or inactivation of ZEP is likely related to the oxidation of specific conserved Cys and/or Met residues. The identification of the targeted amino acids and their importance for ZEP activity is thus important for the detailed understanding of the regulation and function of ZEP.

## Author Contributions

Peter Jahns and Stephanie Bethmann wrote the original draft and reviewed and edited the manuscript.

## Data Availability

Data sharing is not applicable to this article as all newly created data is already contained within this article.
